# Immune responses to Mycobacterial heat shock protein 70 accompany self-reactivity to human BiP in rheumatoid arthritis

**DOI:** 10.1038/srep22486

**Published:** 2016-03-01

**Authors:** Hirofumi Shoda, Norio Hanata, Shuji Sumitomo, Tomohisa Okamura, Keishi Fujio, Kazuhiko Yamamoto

**Affiliations:** 1 Department of Allergy and Rheumatology, Graduate School of Medicine, the University of Tokyo, 7-3-1 Hongo, Bunkyo-ku, Tokyo, Japan

## Abstract

Rheumatoid arthritis (RA) is an autoimmune disease, and a member of human heat shock protein (HSP) 70 protein family, Binding Immunoglobulin Protein (BiP), has been identified as an important autoantigen for T and B cells. We herein focused on Mycobacterial (Myc) HSPs and immune responses to MycHSPs in RA patients. Serum titers of antibodies against MycHSP70 were significantly elevated in RA patients and correlated with serum anti-BiP antibody titers. A MycHSP70-derived HLA-DR4 major epitope was identified using the proliferative capacity of RA PBMCs as an indicator. The major epitope, MycHSP70_287–306_, was located at the corresponding position in the major epitope for human BiP_336–355_, and a strong correlation was found between the proliferation of PBMCs in response to MycHSP70_287–306_ and BiP_336–355_. The immunization of HLA-DR4 transgenic mice with MycHSP70 induced the proliferation of T cells and development of anti-BiP antibodies. In contrast, the oral administration of MycHSP70_287–306_ resulted in the amelioration of collagen-induced arthritis, serum antibody responses, and T cell proliferation. In conclusion, immune responses to MycHSP70 were associated with adaptive immunity against BiP in RA, and could be an important mechanism underlying the development of autoimmunity.

Genetic and environmental factors are causative elements in the development of rheumatoid arthritis (RA). The microbiota is an environmental factor that may contribute to uncontrolled immune responses to self-antigens^1^. Several autoantigens have been reported for RA. Immune responses to citrullinated antigens, such as anti-citrullinated peptide antibodies (ACPAs), have been suggested to play pivotal roles in the pathogenesis of RA. Nevertheless, the precise mechanisms responsible for the breakdown of self-tolerance have not yet been elucidated in detail.

Molecular mimicry is one hypothesis that has been proposed for the development of autoimmunity^2^. The amino acid sequences of some proteins that are necessary for cell homeostasis have been evolutionarily preserved. Immune responses to such bacterial antigens may cross-react and induce immune responses to the corresponding autoantigens. For example, enolase from *Porphyromonas gingivalis* is similar to human alpha-enolase and induces autoimmunity to mammalian alpha-enolase^3^. Vinculin is a membrane-cytoskeletal protein in focal adhesion plaques. van Heemst *et al.* recently reported that citrullinated vinculin is a novel autoantigen for ACPA antibodies^4^. Autoreactive T cells that specifically recognize a DERAA-containing vinculin epitope cross-react with DERAA sequences derived from various pathogens.

The heat shock protein (HSP) family is evolutionarily preserved from prokaryotes to mammals. HSPs are molecular chaperones and are required for cell homeostasis. Autoimmune responses to some HSPs, including Mycobacterial (Myc) HSP65 and Binding Immunoglobulin protein (BiP), a member of the HSP70 family, have been reported in RA, and the induction of tolerance to these HSPs has been investigated as a new therapeutic approach against this disease^5^,^6^. We have shown B cell responses to citrullinated BiP in RA and identified effector and regulatory BiP epitopes for T cells^7^,^8^. Previous studies reported the regulatory effects of MycHSP70 via the production of IL-10 and MycHSP70-derived peptide-specific regulatory T cells in mouse models of arthritis^9^,^10^. Other studies have established several MycHSP70-specific T cell clones with proliferative capacities and IFN-γ production potentials^11^. Therefore, the precise features of MycHSP70-specific T cells in RA remain unclear. The results of the present study revealed a close relationship between immune responses to MycHSP70 and human BiP in RA patients, which could support the importance of Myc and human HSPs in RA immunity.

## Results

### Serum anti-bacterial and human HSP antibodies in RA

**Figure 1:**
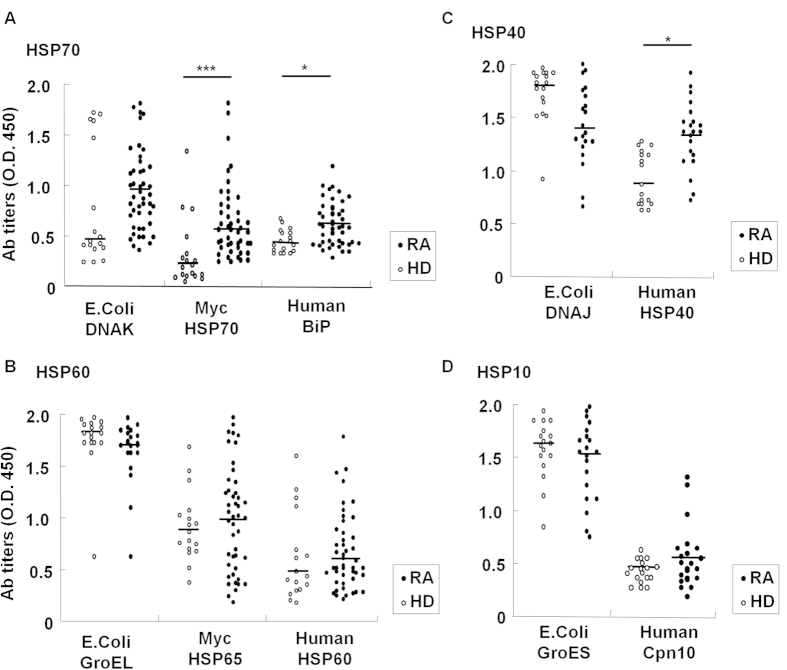
Serum IgG antibody titers to bacterial and human HSPs in RA. (**A–D**) Serum antibody titers to Mycobacterium (Myc) and human HSPs in RA patients and healthy donors (HDs). (**A**) Antibodies to bacterial HSP70 (DNAK) and human BiP. (**B**) Antibodies to bacterial HSP65 (GroEL) and human HSP60. (**C**) Antibodies to bacterial HSP40 (DNAJ) and human HSP40. (**D**) Antibodies to bacterial HSP10 (GroES) and human HSP10 (Cpn10). Horizontal line: median. *p < 0.05, **p < 0.01, ***p < 0.001.

**Figure 2:**
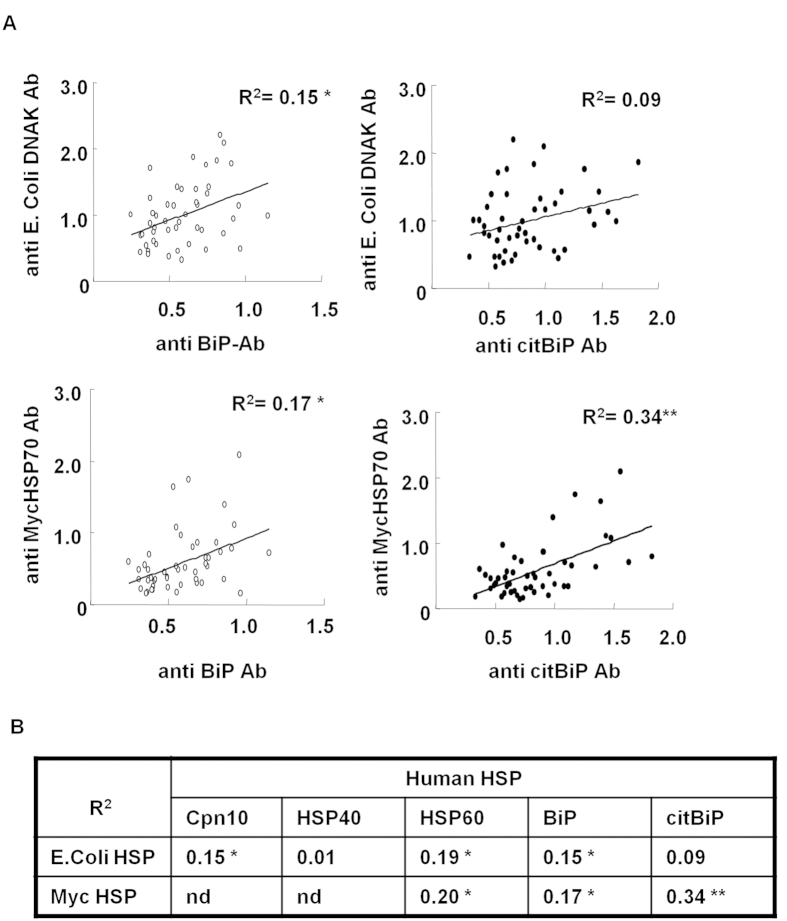
Relationships between anti-HSP antibodies. (**A**) Relationships between serum anti-MycHSP70, anti-human BiP, and citrullinated BiP antibody titers in RA. (**B**) Relationship between serum anti-MycHSP and anti-human HSP antibody titers in RA. nd: no data. *p < 0.05, **p < 0.01.

Serum antibody titers for human and the corresponding bacterial HSPs were measured in RA patients and healthy donors (HDs) (Fig. 1). Cardiovascular disease patients were excluded because of the presence of serum anti-human HSP70 antibodies in these patients^12^. Anti-human BiP antibody titers were significantly higher in RA patients than in HDs (Fig. 1A), whereas serum anti-human HSP60 antibody titers were similar (Fig. 1B). Anti-MycHSP70 antibody titers were also increased in RA sera (Fig. 1A), whereas anti-MycHSP65 antibody titers were not (Fig. 1B). The results obtained for anti-human HSP60 and anti-MycHSP65 antibodies were consistent with previous findings^13^,^14^. Anti-human HSP40 antibody titers were significantly higher in RA patients than in HDs (Fig. 1C), whereas no significant difference was observed in serum anti-human Cpn10 antibody titers (Fig. 1D). As a model of microbial mucosal exposure, we selected *E. coli* HSPs as a control. Although sequence similarity between MycHSPs and *E.coli* HSPs was up to 60%, no significant differences were observed in antibody titers against *E. coli* HSPs between RA patients and HDs (Fig. 1). We then found a correlation between anti-human HSP antibody titers and anti-MycHSP antibody titers (Fig. 2A,B). Anti-MycHSP70 antibody titers and anti-citrullinated BiP antibody titers, in particular, showed a clear positive correlation (Fig. 2A). Anti-MycHSP70 antibody titers were significantly increased in RA patients and were associated with anti-human BiP and citrullinated BiP antibody titers.

### MycHSP70-derived HLA-DR4 epitopes in RA patients

**Figure 3:**
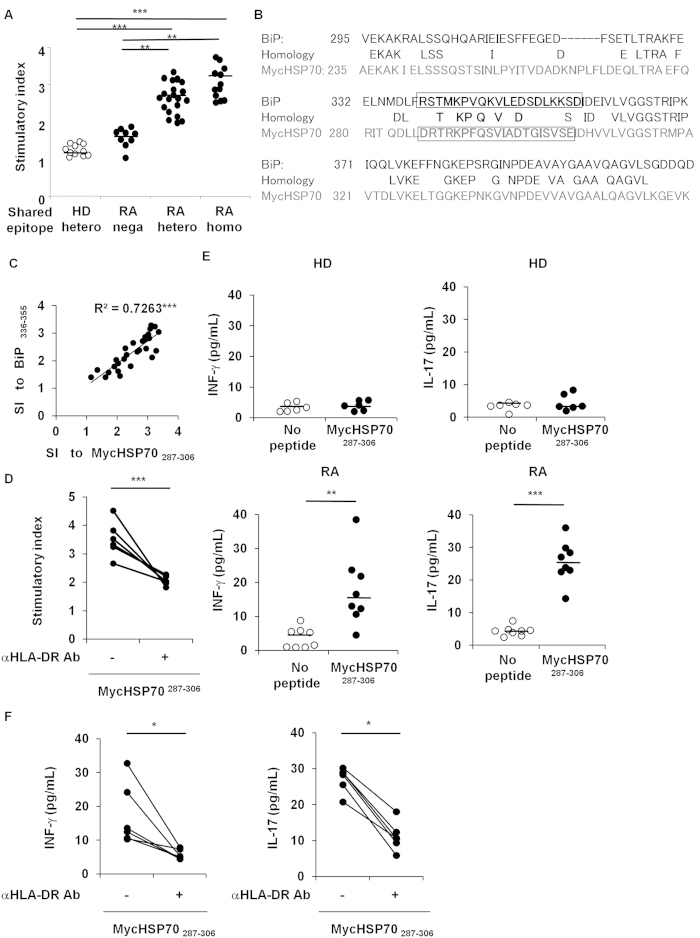
MycHSP70_287–306_ was a HLA-DR4-restricted epitope in RA. (**A**) The proliferation of PBMCs in RA patients (Shared epitope (SE) +/+ n = 12, SE +/− n = 22, SE−/− n = 9) and HDs (SE +/− n = 10) in response to MycHSP70_287–306_. Proliferation was determined by the incorporation of 3-H thymidine. The stimulation index (SI) was calculated using the following equation: (median thymidine uptake to an antigen)/(median thymidine uptake in the absence of an antigen). Horizontal line: the median. (**B**) Amino acid sequences of MycHSP70 (lower lines) and human BiP (upper lines) near the epitopes. Epitope sequences are indicated in bold letters. (**C**) Relationship between MycHSP70_287–306_-induced proliferation and BiP_336–355_-induced proliferation in RA PBMCs (9 patients with SE +/+ and 14 patients with SE +/−). (**D**) Blockade of the MycHSP_287–306_-induced proliferation of PBMCs from HLA-DR4-positive RA patients (n = 6) using an anti-HLA-DR antibody. (**E**) The secretion of MycHSP_287–306_-induced IFN-γ from the PBMCs of HLA-DR4-positive HDs (n = 6) and RA patients (n = 8). (**F**) Blockade of MycHSP_287–306_-induced cytokine secretion from the PBMCs of HLA-DR4-positive RA patients (n = 6) using an anti-HLA-DR antibody. *p < 0.05, **p < 0.01, ***p < 0.001.

In order to detect HLA-DR4-restricted, RA-associated MycHSP70 epitopes, we screened 43 MycHSP70-derived peptides using PBMCs from shared epitope (SE)-positive RA patients (Supplementary Figure S1). Similar to BiP-derived epitopes^8^, at least five peptides induced the proliferation of PBMCs (mean stimulation index >2.5) and, thus, were considered to be candidate peptides for HLA-DR4 epitopes. Of these, MycHSP70_287–306_ (DRTRKPFQSVIADTGISVSE) provoked the highest proliferative responses, which were dependent on the dose of SE in RA patients (Fig. 3A). MycHSP70^287–306^_–_induced proliferation was not observed in HLA-DR4-positive HD PBMCs (Fig. 3A). MycHSP70 and BiP shared 50% amino acid sequence similarity and MycHSP70_287–306_ located at the corresponding position of the major epitope for human BiP, BiP_336–355_ (RSTMKPVQKVLEDSDLKKSD), which we previously identified as a RA-related HLA-DR4 effector epitope (Fig. 3B)^8^. PBMC proliferation to MycHSP70_287–306_ was significantly dependent on the SE dose, and this dependency was similar to our previous findings for BiP_336–355_^8^. MycHSP70_287–306–_induced proliferation correlated with BiP_336–355_-induced proliferation in RA (Fig. 3C). These proliferation responses to MycHSP70_287–306_ peptides were clearly inhibited by the blockade of HLA-DR (Fig. 3D). Regarding the production of cytokines, the secretion of interferon (IFN)-γ and interleukin (IL)-17 was induced by MycHSP70_287–306_ peptides and was also blocked by the anti-HLA-DR antibody (Fig. 3E,F).

**Figure 4:**
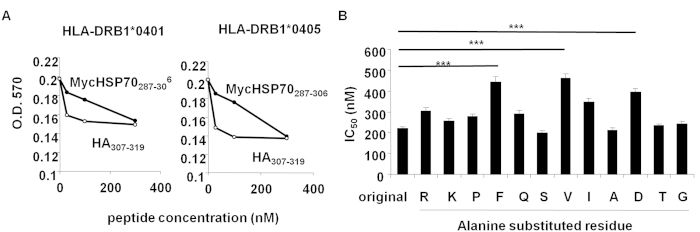
HLA-DR4 binding assay. (**A**) Binding activities between a HLA-DRB1*0401 and 0405 molecule and MycHSP70_287–306_. Binding activities were estimated by the inhibition of peroxidase-labeled HA_307–318_ peptide binding to a HLA-DRB1 molecule. (**B**) Binding of MycHSP70_287–306_ with alanine-substituted peptides to HLA-DRB1*0405 molecules. A MycHSP70_287–306_-derived peptide in which an amino residue was substituted by an alanine residue. Inhibitory concentration 50% (IC_50_) was used as a measure of binding activity. *p < 0.05, **p < 0.01, ***p < 0.001.

A binding assay showed that MycHSP70_287–306_ bound to the HLA-DRB1*0401 and 0405 molecule (Fig. 4A). Alanine scanning of this peptide showed that the substitution of F293 (P1), V296 (P4), and D299 (P7) significantly decreased its binding ability (Fig. 4B). These results demonstrated that MycHSP70_287–306_ is an immunogenic HLA-DR4 epitope in RA patients.

### Immunization of MycHSP70 broke self-tolerance in mice

**Figure 5:**
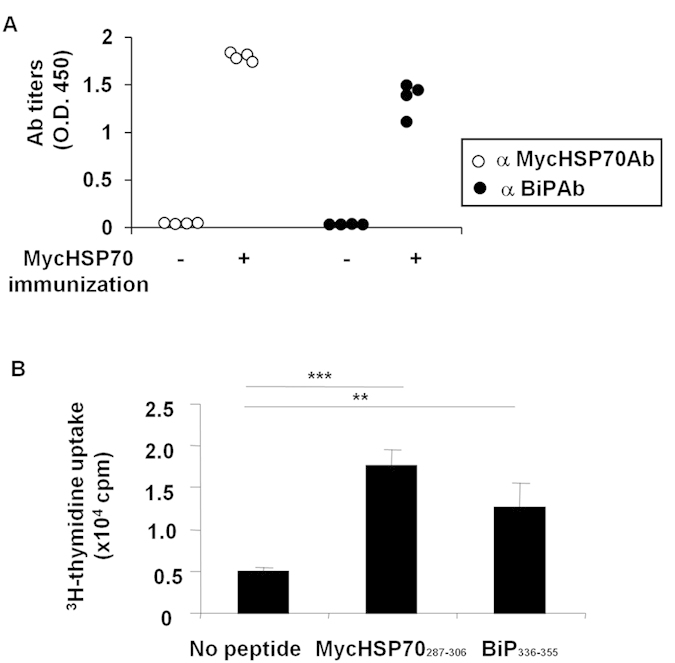
Immunization of HLA-DR4 transgenic mice with MycHSP70. (**A**) HLA-DR4 transgenic mice were immunized with recombinant MycHSP70. Serum anti-MycHSP70 and anti-mouse BiP antibody titers were measured in the 21 days following the first immunization. (**B**) Splenic CD4+ T cell proliferation from MycHSP70-immunized HLA-DR4 transgenic mice in response to MycHSP70_287–306_ and BiP_336–355_ epitopes. Proliferation was determined by the incorporation of 3-H thymidine. Data were representative of at least three independent trials. ***p < 0.001.

We next addressed adaptive immunity induced by MycHSP70_287–306_ *in vivo*. The immunization of HLA-DR4 transgenic mice with the MycHSP70 protein led to an elevation in serum antibody titers to MycHSP70 and BiP (Fig. 5A). Furthermore, significant CD4^+^ T cell proliferation was induced in response to the MycHSP70_287–306_ peptide, providing additional evidence that the MycHSP70_287–306_ peptide was an HLA-DR4 epitope (Fig. 5B). The significant proliferation of CD4^+^ T cells in response to the BiP_336–355_ peptide was also observed (Fig. 5B). These results indicate that immune responses to MycHSP70 induce the loss of self-tolerance to BiP.

### Oral administration of the MycHSP70 epitope ameliorated collagen-induced arthritis

**Figure 6:**
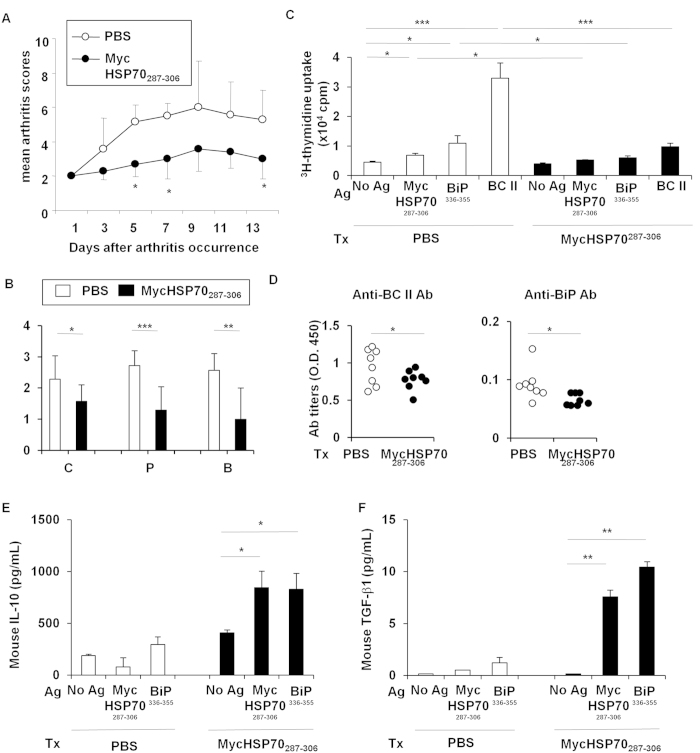
Oral tolerance of MycHSP70_287–306_ peptides in a collagen-induced arthritis model. (**A,B**) The MycHSP70_287–306_ peptide or control (PBS alone) was orally administered to bovine type II collagen-immunized DBA/1 J mice after the occurrence of CIA for 5 continuous days (n = 12). Mice were sacrificed on day 35. Arthritis scores were measured by finger and paw swelling, and histological scores were measured by cell infiltration (**C**), pannus formation (P), and bone destruction (**B**). (**C**) Splenic CD4^+^ T cell proliferation from CIA-induced DBA/1 J mice in response to a recall antigen (Ag) stimulation (MycHSP70_287–306_ and BiP_336–355_ epitopes, and heat-denatured bovine type II collagen (BC II)). Proliferation was determined by^3^-H thymidine incorporation. (**D**) Serum anti-BiP and anti-BC II antibody titers were measured by ELISA. (**E**,**F**) Mouse IL-10 and TGF-beta1 concentrations in the splenic CD4^+^ T cell culture were measured by ELISA. Data were representative of at least three independent trials. *p < 0.05, **p < 0.01, ***p < 0.001.

We also examined the therapeutic effects of the oral administration of the MycHSP70_287–306_ peptide on collagen-induced arthritis (CIA) in DBA/1 J mice. The oral administration of the MycHSP70_287–306_ peptide significantly improved the development of arthritis and histological scores in CIA (Fig. 6A,B). The proliferation of CD4^+^ T cells in response to the MycHSP70_287–306_ and BiP_336–355_ peptides was reduced by the orally administered MycHSP70_287–306_ peptide (Fig. 6C). The oral administration of the MycHSP70_287–306_ peptide also suppressed bovine type II collagen-specific T cell proliferation (Fig. 6C) and pro-inflammatory cytokine secretion from CD4^+^ T cells (Supplementary Figure S2). Serum anti-BiP and anti-BC II antibody titers were significantly decreased in MycHSP70_287–306_-treated mice (Fig. 6D). Furthermore, the secretion of IL-10 and TGF-β1 from CD4^+^ T cells was induced by MycHSP70_287–306_ and BiP_336–355_ recall stimulations in MycHSP70_287–306_-treated mice (Fig. 6E,F). The amount of cytokines released in response to HSP70 epitopes was previously reported to be lower than that from orally tolerized TCR-transgenic CD4^+^ T cells^15^,^16^. This finding suggests that the frequencies of MycHSP70_287–306_ and BiP_336–355_-specific T cells were limited in this model. The frequency of CD4^+^ CD25^+^ Foxp3^+^ regulatory T cells was increased by the oral administration of the MycHSP70_287–306_ peptide to CIA mice (Supplementary Figure S2). These results indicate that the induction of tolerance to MycHSP70 ameliorated inflammatory arthritis and tolerance to autoantigens, including BiP.

## Discussion

Environmental factors account for half of the causes of seropositive RA^17^. With the exception of a smoking habit, most environmental risks remain unknown because difficulties are associated with following environmental exposure from birth to the age of arthritis onset. Since a T cell clone specific to the Mycobacterial-derived epitope, MycHSP65_180–188_, has arthritogenic capacity, Mycobacterial exposure was presented as a potential candidate environmental risk factor for RA^6^. RA has been shown to increase the risk of Mycobacterial infection independent of immunosuppressive therapies^18^. Nevertheless, evidence for the relationship between Mycobacterial infection with the pathogenesis and exacerbation of RA is limited. We herein demonstrated that T and B cell responses to MycHSP70 were significantly enhanced in RA, which suggests Mycobacterial exposure in RA patients. Moreover, PBMC proliferative responses to MycHSP70_287–306_ and BiP_336–355_ were closely associated. T cell repertoire analysis demonstrated the clonal expansion of T cells in RA, and the increase observed in proliferation against these epitopes could be partially explained by RA PBMCs containing more antigen-experienced clones^19^. Moreover, we previously reported that BiP_336–355_-induced PBMC proliferation correlated with serum anti-BiP antibody titers. The immunological basis for this correlation may be important for understanding the pathogenesis of RA.

MycHSP70 and BiP share 50% of their amino acid sequences, and MycHSP70_287–306_ is located at a position corresponding to that of the BiP-derived HLA-DR4 effector epitope. We previously identified two major BiP-derived HLA-DR4 epitopes in RA. One (BiP_336–355_) was recognized by effector T cells and the other (BiP_456–475_) by IL-10-secreting regulatory T cells^8^. MycHSP70_287–306_ strongly induces proliferation and is regarded as an effector epitope. MycHSP70_287–306_ and BiP_336–355_ share 50% of the amino acids in their core sequences, which bind HLA-DR pockets. They also share the P4V residue, which is regarded as the most important amino acid for HLA-DR4 binding^20^. This sequence similarity could contribute to the breakdown of T cell tolerance to self BiP in RA, and also suggests the role of molecular mimicry; however, we were unable to exclude the contribution of epitope spreading. One possibility is that the same clone recognizes MycHSP70_287–306_ and BiP_336–355_. A vinculin-specific TCR has been shown to recognize DERAA-containing epitopes from three bacterial species, *Campylobacter coli, Lactobacillus curvatus*, *and L. sakei*^4^. A second possibility is epitope spreading from the Myc antigen to a self-antigen that shares similarities via recognition by different T cell clones. Epitope spreading is described as the diversification of T cell activation from one epitope to non-cross-reactive autoantigen-derived epitopes and is induced during chronic inflammation. T cell epitope spreading has mainly been examined in mouse models of multiple sclerosis^21^,^22^. The findings obtained suggest that intermolecular and intramolecular epitope spreading occurs as a result of inflammation and tissue damage. According to this hypothesis, inflammation induced by MycHSP70-specific T cells could up-regulate the expression of BiP and BiP-specific T cell activation. These possibilities warrant further study.

Mycobacterial species, particularly non-tuberculous mycobacteria, live in the soil and water, and a large number of individuals are continuously exposed to several mycobacteria in daily life^23^. Most of these bacteria are considered to be non-pathogenic for humans. However, difficulties are associated with detecting and identifying these bacteria due to their slow growth rates. Next generation sequencing of 16S rRNA in microbiota has provided new evidence for the “silent” exposure of humans to Mycobacterial species^24^. The clear correlation between serum anti-MycHSP70 antibody titers and anti-citrullinated BiP antibody titers indicates that a relationship exists between immune responses to mycobacteria with ACPA formation. Mycobacteria in the lungs have been shown to play a role in the pathogenesis of RA because the lungs are a candidate initiator organ in which the triggering of specific immunity occurs in RA^24^. However, most RA patients have no history of Mycobacterial infection. Since immune responses to mycobacteria were activated in RA patients, our results imply the clinical importance of subclinical exposure to mycobacteria and its association with the pathogenesis of RA. If it is possible to determine the route of exposure, vaccination or tolerance induction to mycobacteria may become a new strategy for RA prevention and therapy. Since the oral administration of the MycHSP70_287–306_ peptide improved experimental arthritis and suppressed autoreactive T cell reactions, the presence of mycobacteria in the gut, but not in the lungs, may regulate autoimmunity against self-HSPs.

As a limitation of this study, the patients who participated took steroids and/or immunosuppressive drugs, which may have affected their T and B cell functions. Even though these potentially immunosuppressive drugs were used, increased immune responses were observed in RA patients.

In conclusion, we herein demonstrated the presence of T and B cell responses to MycHSP70 in RA. We identified a new effector T cell epitope derived from MycHSP70, and PBMC proliferation induced by the MycHSP70-derived epitope correlated with that induced by the BiP-derived epitope in RA. The sequence similarity between MycHSP70 and human BiP at the T cell epitope level supports a link between Mycobacterial exposure and the breakdown of tolerance to BiP in RA patients. We also demonstrated the potential of the therapeutic application of these epitopes. The induction of tolerance to the MycHSP70-derived epitope could regulate immune responses to self BiP and arthritis, and, in the present study, we provided evidence to suggest that the induction of tolerance to microbial epitopes induces tolerance to related self-antigens. This system may be a suitable model for understanding how autoimmune responses develop in the pathogenesis of RA, and may lead to new therapeutic strategies for RA.

## Materials and Methods

### Patients and HLA-DRB1 typing

Blood samples were obtained from HDs (n = 20) and RA patients without cardiovascular diseases (n = 48). Demographic data are shown in Table S2. We included RA patients who fulfilled the classification criteria of the 2010 American College of Rheumatology (ACR)/European League Against Rheumatism^25^. Although some established RA patients had been diagnosed by the 1987 ACR criteria, all of them also met the 2010 criteria. No donor had a previous history of active tuberculosis infection. HLA-DRB1 genotyping was performed in accordance with a previous study^26^.Written informed consent was obtained from all subjects before samples were taken. This study was approved by the Ethical Committee of the University of Tokyo Hospital. The methods were performed in accordance with the approved guidelines.

### Proteins

Human recombinant BiP, HSP60, HSP40, and Cpn10 and recombinant MycHSP65 and MycHSP70 were purchased from Enzo Life Sciences. *E. coli* recombinant DNAK, GroES, DNAJ, and GroEL were purchased from ProSpec-Tany TechnoGene. The recombinant MycHSP70 protein (NCBI GenBank AF2122/97) was produced for the mouse immunization study using the pET Directional TOPO Expression Kit (Life Technologies) and purified by the Ni-NTA Fast Start Kit (QIAGEN) according to the manufacturer’s procedures. The preparation of citrullinated BiP was described previously^7^. Endotoxin was removed by passing through a Detoxi-Gel AffinityPak prepared column (Pierce).

### Antibody measurement

Serum antibodies were measured by ELISA as previously reported^7^. Briefly, 96-well plates (Thermo Fisher Scientific) were coated with 5 μg/mL of each antigen and dissolved in 0.1 M NaHCO_3_. Diluted samples (100x) were applied, followed by goat anti-human IgG-horseradish peroxidase (HRP) (5000:1,Vector) or goat anti-mouse IgG-HRP (5000:1, Life Technologies) detection antibodies and TMB solution (KPL) for development.

### Peptide synthesis

MycHSP70-derived peptides were designed to be 20-mers long and overlap one another by at least 5-mers (Supplementary Table S1) (Operon Biotechnology). The purity of each peptide was greater than 70%. Alanine-substituted peptides, which were derived from MycHSP_287–306_ and had one amino acid substituted to alanine, were prepared for the binding assay.

### Human PBMC culture

PBMCs (1 × 10^6^/mL) isolated with Ficoll-Paque (GE Healthcare) were cultured in the presence or absence of each peptide (10 μg/mL) with RPMI 1640 medium supplemented with 10% human serum, 2 mM L-glutamine, 100 U/ml penicillin, and 100 μg/ml streptomycin in a humidified incubator at 37 °C, 5% CO_2_, for 96 hours. Cells were incubated for 18 h following a ^3^H-thymidine pulse into each well and harvested using a semiautomated sample harvester. Counts per minute were measured with a scintillation counter. In some experiments, the culture supernatants were collected after a 72-hour incubation and cytokine concentrations were measured in the culture supernatants by a human IFN-γ high sensitivity ELISA kit and human IL-17 high sensitivity ELISA kit (eBioscience). In blocking experiments, 10 μg/mL of an anti-HLA DR monoclonal antibody (L243) (Biolegend, San Diego, CA, USA) was added.

### Peptide binding assay

The procedure to assess peptide binding to HLA-DRB1*0401 and 0405 was described in a previous study^8^. In brief, HLA-DRA1B1*0401 and 0405 was cloned and transfected into S2 *Drosophila* cells. Soluble DR4 molecules in the supernatant were collected and purified through an affinity column coupled with the anti-DR antibody LB3.1. In the peptide binding assay, a 40 nM solution of purified DR4 was incubated at 37 °C for 4 h with the HA_307–319_ peptide (0.5 nM), which had been labeled at the N terminus with biotin. The indicated concentrations of the peptides were added as competitors to HA peptide binding. Bound peptides were separated from free peptides by immobilizing the DR molecules on microtiter plates coated with LB3.1. The antibody was coated to the plate by the overnight incubation of a 5 μg/mL solution at 4 °C. Bound biotinylated peptides were detected by an incubation with streptavidin-alkaline phosphatase. An automatic microplate reader (Bio-rad 550, Bio-rad) was used to measure optical density.

### HLA-DR4 transgenic mice

HLA-DR4 transgenic mice were obtained from Taconic, immunized twice (day 0, 14) with 100 μg of MycHSP70 with the same volume of complete Freund’s adjuvant subcutaneously into the tails, and sacrificed on day 21. MACS-purified CD4^+^ T cells (1 × 10^5^/mL) were cultured with irradiated splenocytes (1 × 10^6^/mL) and an antigen (10 μg/mL) in RPMI 1640 medium supplemented with 5% FCS, 2 mM L-glutamine, 100 U/ml penicillin, 100 μg/ml streptomycin, and 5 × 10^−5^ M 2-mercaptoethanol in a humidified incubator at 37 °C, 5% CO_2_, for 72 h. ^3^H-thymidine was pulsed to each well, cells were harvested after an 18-hour incubation, and analyzed with a scintillation counter.

### CIA

CIA in the background of DBA/1 J female mice (SLC Japan) was performed and arthritis scores were acquired as previously described^7^. In the therapeutic experiments, mice were administered 200 μg of MycHSP_287–306_ peptides orally for 5 continuous days following the onset of arthritis. Mice were sacrificed on day 35, and the CD4^+^ T cell culture was performed as described above. Mouse cytokine concentrations in the supernatants were measured using a mouse IL-10 ELISA kit and mouse/human TGF-beta 1 ELISA kit (eBioscience) according to the manufacturer’s protocol. Tissue samples were embedded in paraffin wax after 10% formaldehyde fixation and decalcification. Sections were stained with hematoxylin and eosin. Synovial tissues were graded by mononuclear cell infiltration, pannus formation, and cartilage erosion, as described previously^7^. All animal experiments were conducted in accordance with Institutional and National Guidelines, and the protocols were approved by the Ethical Committee on Animal Experiments of the University of Tokyo.

### Statistical analysis

Data were expressed as the median ± SEM. Differences were compared with the Mann-Whitney U test. Comparisons of more than three groups were performed using a one-way analysis of variance followed by a Bonferroni multiple comparison. Correlations were evaluated using Spearman’s rank correlation coefficient. *P* values less than 0.05 were considered significant.

## Additional Information

**How to cite this article**: Shoda, H. *et al.* Immune responses to Mycobacterial heat shock protein 70 accompany self-reactivity to human BiP in rheumatoid arthritis. *Sci. Rep.* **6**, 22486; doi: 10.1038/srep22486 (2016).

## Supplementary Material

Supplementary Information
