# Supplementary material for: Immune responses to Mycobacterial heat shock protein 70 accompany self-reactivity to human BiP in rheumatoid arthritis
Source: Sci Rep. 2016 Mar 1;6:22486. doi: 10.1038/srep22486 (PMC4772543; doi:10.1038/srep22486)
Supplement: Supplementary Information [file srep22486-s1.pdf]

Supplementary information

Title: Immune responses to Mycobacterium heat shock protein 70 accompany self-reactivity to human BiP in rheumatoid arthritis.

Authors: Hirofumi Shoda, Norio Hanata, Shuji Sumitomo, Tomohisa Okamura, Keishi Fujio, Kazuhiko Yamamoto

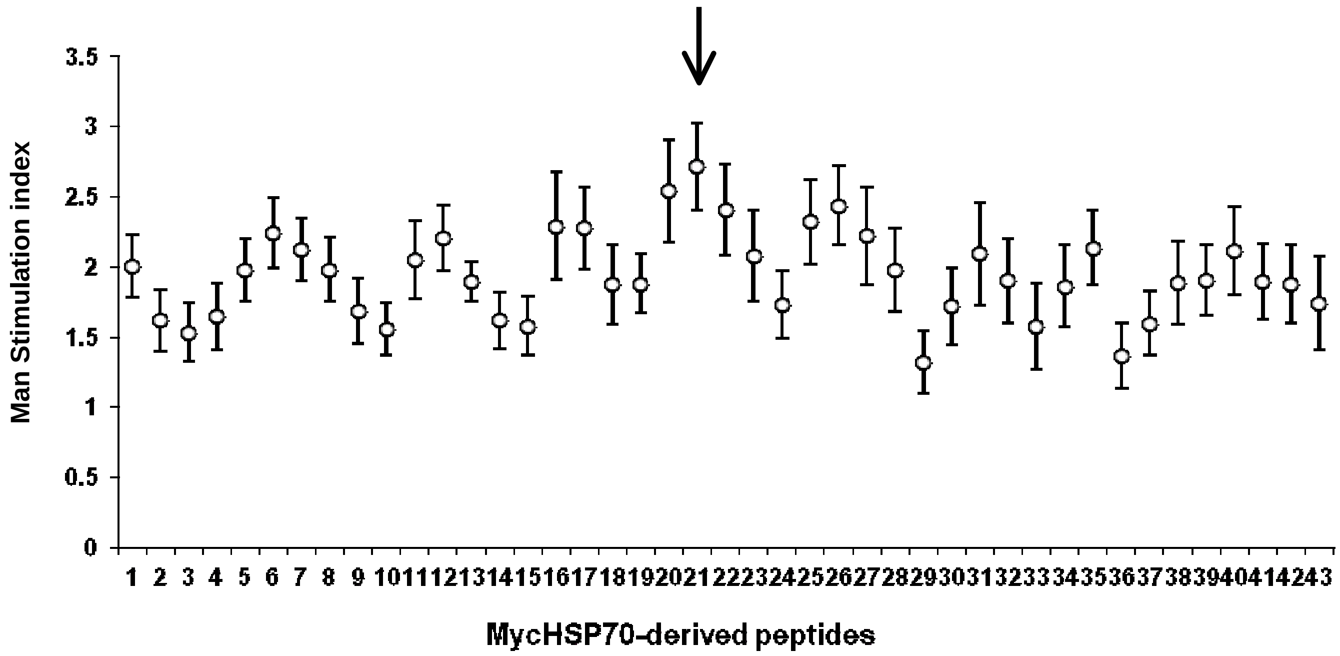

Man Stimulation index
3.5
3
2.5
2
1.5
1
0.5
0
1 2 3 4 5 6 7 8 9 10111213141516171819202122232425262728293031323334353637383940414243
MycHSP70-derived peptides

A

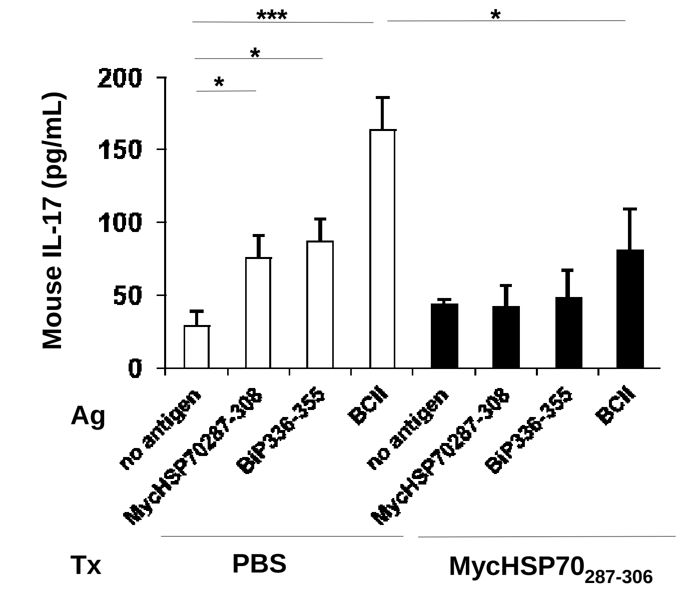
***
*
*
*
200
150
100
50
0
Mouse IL-17 (pg/mL)
Ag
no antigen
MycHSP70287-308
BiP336-355
BCII
no antigen
MycHSP70287-308
BiP336-355
BCII
Tx
PBS
MycHSP70287-306

B

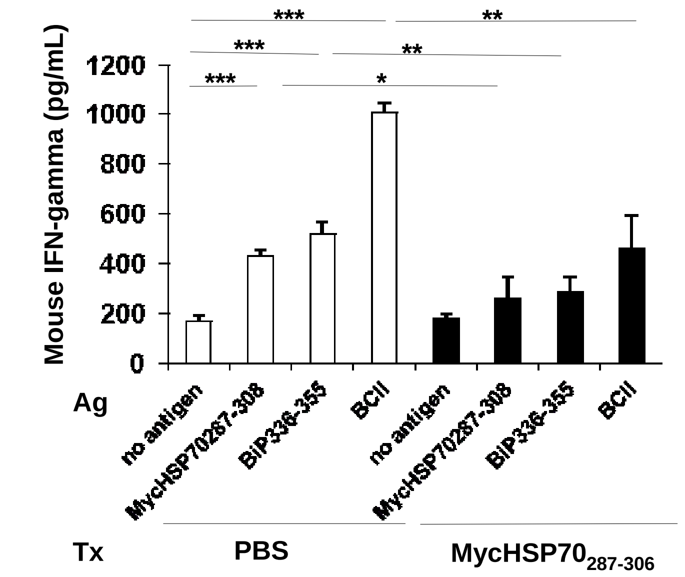
***
**
***
**
***
*
1200
1000
800
600
400
200
0
Mouse IFN-gamma (pg/mL)
Ag
no antigen
MycHSP70287-308
BiP336-355
BCII
no antigen
MycHSP70287-308
BiP336-355
BCII
Tx
PBS
MycHSP70287-306

C

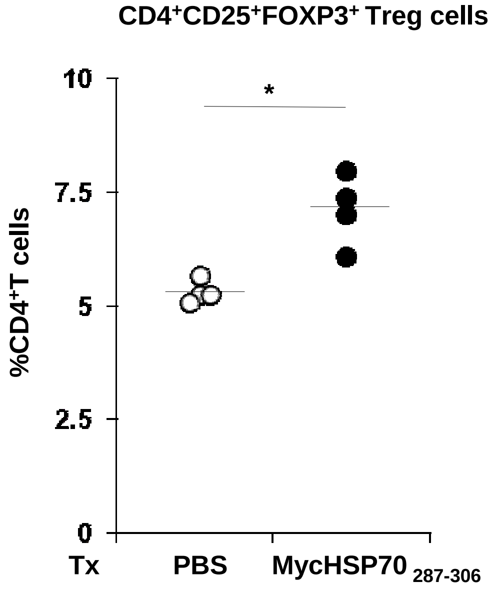
CD4+CD25+FOXP3+ Treg cells
*
10
7.5
5
2.5
0
%CD4+T cells
Tx
PBS
MycHSP70 287-306

Supplementary Table S1

	RA	HD
N	48	20
Age	55.2 ± 20.1	46.1 ± 14.8
Sex (female, male)	41, 7	15, 5
Disease duration (y)	10.8 ± 9.5	0
Stage	2.63 ± 1.25	
Class	1.55 ± 0.63	
RF positivity (%)	70.8	0
ACPA positivity (%)	87.5	0
CRP (mg/dL)	1.07 ± 1.00	
ESR (mm/hr)	40.3 ± 21.7	
DAS28-4CRP	3.37 ± 0.80	
DAS28-4ESR	4.11 ± 0.86	
Methotrexate (mg/week)	8.7 ± 2.3	
Predonisolone (mg/day)	3.5 ± 3.6	
Biologics user (%)	IFX 4.2%<br>ETN 6.2%	

Supplementary Table S2

Number	Amino acid sequences	Position
MycHSP70-1	MARAVGIDLGTTNSVVSVLE	1-20
MycHSP70-2	VSVLEGGDPVVVANSEGSRT	16-35
MycHSP70-3	EGSRTTPSIVAFARNGEVLV	31-50
MycHSP70-4	GEVLVGQPAKNQAVTNVDRT	46-65
MycHSP70-5	NVDRTVRSVKRHMGSDWSIE	61-80
MycHSP70-6	HMGSDWSIEIDGKKYTAPEI	72-91
MycHSP70-7	TAPEI SARILMKLKRDAEAY	87-106
MycHSP70-8	DAEAY LGEDITDAVITTPAY	102-112
MycHSP70-9	TTPAYFNDAQRQATKDAGQI	117-136
MycHSP70-10	DAGQIAGLNVLRIVNEPTAA	132-151
MycHSP70-11	EPTAAALAYGLDKGEKEQRI	147-166
MycHSP70-12	KEQRILVFDLGGGTFDVSLL	162-181
MycHSP70-13	DVSLLEIGEGVVEVRATSGD	177-196
MycHSP70-14	ATSGDNHLGGDDWDQRVVDW	192-211
MycHSP70-15	RVVDW LVDKFKGTSGIDLTK	207-226

MycHSP70-16	IDLTKDKMAMQRLREAAEKA	222-241
MycHSP70-17	AAEKAKIELSSSQSTSINLP	237-256
MycHSP70-18	KIELSSSQST SINLP YITVD	237-256
MycHSP70-19	YITVD ADKNP LFLDE QLTRA	242-261
MycHSP70-20	QLTRAEFQRITQDLLDRTRK	257-276
MycHSP70-21	DRTRKPFQSVIADTGISVSE	287-306
MycHSP70-22	ISVSEIDHVVLVGGSTRMPA	302-321
MycHSP70-23	TRMPAVTDLVKELTGGKEPN	317-336
MycHSP70-24	GKEPNKGVNPDEVVAVGAAL	332-351
MycHSP70-25	VGAALQAGVLKGEVKDVLLL	347-366
MycHSP70-26	VKDVLLLDVTPLSLGIETKG	360-379
MycHSP70-27	IETKGGVMTRLIERNTTIPT	375-394
MycHSP70-28	TTIPTKRSETFTTADDNQPS	390-409
MycHSP70-29	DNQPSVQIQVYQGEREIAAH	405-424
MycHSP70-30	EIAAHNKLLGSFELTGIPPA	420-439
MycHSP70-31	GIPPAPRGIPQIEVTFDIDA	435-454
MycHSP70-32	FDIDANGIVHVTAKDKGTGK	450-469
MycHSP70-33	KGTGKENTIRIQEGSGLSKE	465-484

MycHSP70-34	SGLSKEDIDRMIKDAEAHAE	479-498
MycHSP70-35	EAHAEEDRKRREEADVRNQA	494-513
MycHSP70-36	VRNQAETLVYQTEKFVKEQR	509-528
MycHSP70-37	VKEQREAEGGSKVPEDTLNK	524-543
MycHSP70-38	DTLNKVDAAVAEAKAALGGS	539-558
MycHSP70-39	ALGGSDISAIKSAMEKLGQE	554-573
MycHSP70-40	KLGQESQALGQAIYEAAQAA	569-588
MycHSP70-41	AAQAASQATGAAHPGGEPGG	584-603
MycHSP70-42	GEPGGAHPGSADDVVDAEVV	599-618
MycHSP70-43	PGSADDVVDAEVVDDGREAK	606-625

Supplementary Figure S1

Proliferation of PBMCs from HLA-DR4-positive RA patients (n = 20) in response to MycHSP70-derived 20-mer peptides (Supplementary Table 2). Stimulation index (S.I.) indicated the proliferation of PBMC from RA patients cultured in the presence of each peptide (10 μg/mL) for 96 hours, which were analyzed by ^{3}H-thymidine uptake. Black arrow indicates the position of MycHSP70 $_{287\text{-}306}$.

Supplementary Figure S2

Oral tolerance of MycHSP70$_{287\text{-}306}$ peptides on collagen-induced arthritis (CIA) model. The experimental protocol was the same as Figure 6. (A)(B)Mouse IL-17 and IFN-gamma concentrations in the splenic CD4^{+} T cell culture was measured by ELISA (Mouse cytokine ELISA ready-set-go, eBioscience) according to the manufacture’s protocol. (C) The frequencies of splenic regulatory T cells in CIA (n=4, each). FACS analysis was performed by FACS Vantage (BD Bioscience). Cells were stained by anti-mouse CD4 APC antibody, anti-mouse CD25 FITC antibody, FOXP3 staining was performed using anti-mouse Foxp3 PE antibody (eBioscience) by using

Fixation/Permeabilization concentrate and diluent (eBioscience) (eBioscience) according to the manufacture's protocol. *: $p < 0.05$, **:$p<0.01$, ***: $p < 0.001$.

Supplementary Table S1

List of MycHSP70-derived peptides. MycHSP70-derived peptides were designed to be 20-mers long and overlap one another by at least 5-mers.

Supplementary Table S2

Summary of the characteristics of RA patients and healthy donors.
